# Model of Magnetically Shielded Ferrite-Cored Eddy Current Sensor

**DOI:** 10.3390/s22010326

**Published:** 2022-01-02

**Authors:** Darko Vasić, Ivan Rep, Dorijan Špikić, Matija Kekelj

**Affiliations:** 1 Faculty of Electrical Engineering and Computing, University of Zagreb, 10000 Zagreb, Croatia; ivan.rep@fer.hr (I.R.); dorijan.spikic@fer.hr (D.Š.); 2 INETEC Institute for Nuclear Technology, 10250 Zagreb, Croatia; matija.kekelj@inetec.hr

**Keywords:** ferrite core, magnetic shield, eddy current, probe, coil, truncated domain, eigenfunction expansion

## Abstract

Computationally fast electromagnetic models of eddy current sensors are required in model-based measurements, machine interpretation approaches or in the sensor design phase. If a sensor geometry allows it, the analytical approach to the modeling has significant advantages in comparison to numerical methods, most notably less demanding implementation and faster computation. In this paper, we studied an eddy current sensor consisting of a transmitter coil with a finitely long I ferrite core, which was screened with a finitely thick magnetic shield. The sensor was placed above a conductive and magnetic half-layer. We used vector magnetic potential formulation of the problem with a truncated region eigenfunction expansion, and obtained expressions for the transmitter coil impedance and magnetic potential in all subdomains. The modeling results are in excellent agreement with the results using the finite element method. The model was also compared with the impedance measurement in the frequency range from 5 kHz to 100 kHz and the agreement is within 3% for the resistance change due to the presence of the half-layer and 1% for the inductance change. The presented model can be used for measurement of properties of metallic objects, sensor lift-off or nonconductive coating thickness.

## 1. Introduction

Eddy current sensors (probes) respond to changes in the material properties (electrical conductivity, magnetic permeability), geometry or position of the nearby electrically conductive, usually metallic, objects [1,2]. Their applications include nondestructive testing for defects and material changes in objects, such as heat exchangers in nuclear plants, oil well casings, pipelines or aircraft hulls [3,4,5]; measurement of the distance to such objects or their dimensions [6,7]; and material characterization [8,9,10]. Eddy current sensors offer detection of very small surface defects, measurement of dimensions or distances in the micrometer range, insensitivity to nonconductive dirt, such as oil, water or dust, and robustness to the environmental factors such as pressure, temperature or stress. However, absolute measurement using eddy current sensors can be hard to achieve leading to the need for calibration, and human or machine analysis and interpretation of the sensor signals [11,12,13].

Traditionally, an eddy current sensor consists of a transmitter coil that generates a magnetic field and induces the eddy currents in the conductive material [1]. Receiver coils pick up the resultant magnetic field due to the defect and changes in the dimensions and material properties. Receiver coils can be replaced by Hall, magnetoresistive or even super-conducting quantum interference devices (SQUID) sensors [14]. However, the overall characteristics of the eddy current sensors are primarily determined by the excitation, geometry and construction of the transmitter coil.

Fast electromagnetic models of transmitter coils are required in model-based measurements, machine interpretation approach or in parametric and sensitivity analyses in the sensor design phase. When it comes to more complicated sensor geometries, analytical electromagnetic models are not as versatile as numerical ones, e.g., finite-element or boundary-element models. However, if a real sensor and object under test can be approximated by a geometry that is simple enough to be dealt with analytically, then one can expect less demanding numerical implementation and higher computational speed in comparison to fully numerical approaches. More complicated geometries can be treated using hybrid approaches in which the analytical models are used for calculation of the incidence fields and numerical methods for calculation of the fields scattered from the small changes in the tested object [15].

Typically with eddy current sensors, the transmitter coil has a ferrite core to increase the inductance, sensitivity and signal-to-noise ratio or to achieve the desired magnetic field distribution. Since the seminal work by Dodd and Deeds [16], who studied air-cored transmitter coils in planar and cylindrical geometries (layers, rods, tubes), a number of more complicated geometries have been analyzed using the truncated region eigenfunction expansion approach (TREE) [17]. This includes ferrite-cored transmitter coils, which previously required numerical or empirical approaches. In a paper that laid out the modeling path followed by other authors, Theodoulidis analyzed the coil with the cylindrical finite-length ferrite core that was positioned above a half space made of two nonmagnetic and conductive layers [18]. This analysis was extended to a half space made of multiple magnetic and conductive layers by Lu et al. in [19]. Bayani et al., in [20], developed the model of a cup-cored coil above a nonmagnetic conductive double layered half space. In all three studies, the models were verified experimentally and the relative error between the theoretical results and measurements were on average between 0.5% and 2.5%. Sakkaki and Bayani, in [21], modeled an E-cored coil above a conductive double layer, and they numerically verified the model by comparing it to a finite element model using Comsol. Tytko and Dziczkowski, in [22,23,24], modeled the I-cored and E-cored coils in the presence of the layered medium with a hole, and they verified their work using a Comsol-based finite element model. Zhu et al., in [25], analyzed an I-cored coil screened with a thin sheet of material with infinite permeability.

**Figure 1:**
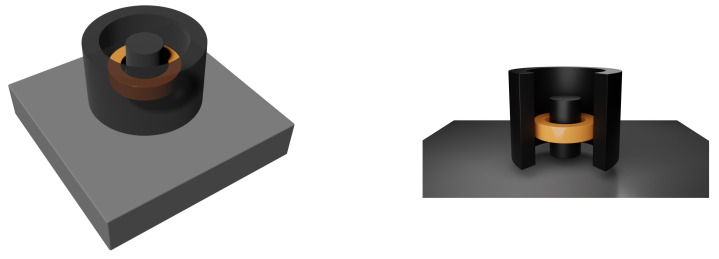
The sensor above the plate. The shield is shown as transparent/cut to show the sensor inside.

**Figure 2:**
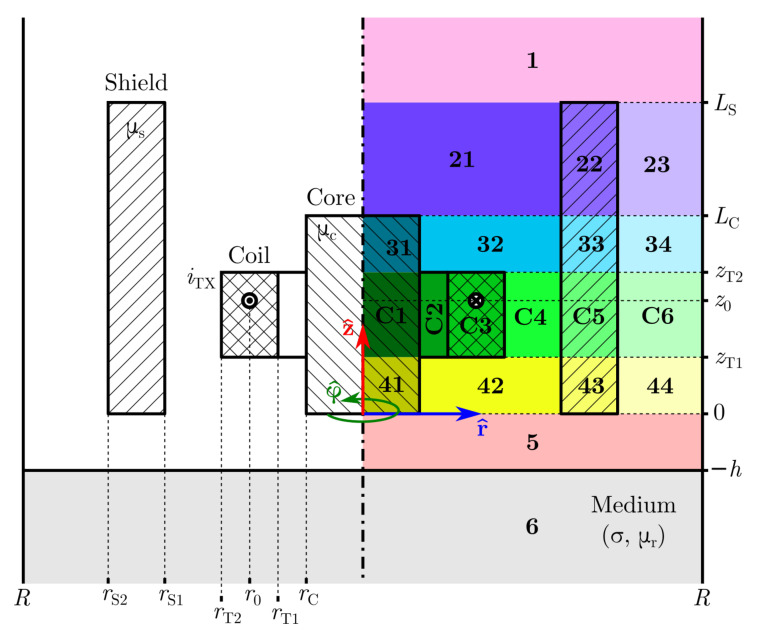
A ferrite-cored coil screened with a ferrite shield above magnetic and conductive half space. A filamentary coil is also shown. For better clarity, the subregions of a region are indicated by colors with the same hue value but different lightness. In the case of the filamentary coil, region C is not present and the boundary between regions 3 and 4 is at $z=z_{0}$.

In this paper, we studied a coil with a finitely long I core and screened with a finitely thick magnetic shield placed above a conductive and magnetic half-layer using the TREE approach. Figure 1 and Figure 2 depict the geometry in more detail. We made no assumptions regarding the shield thickness and the permeability of the shield and the core. The open upper side of the shield allows for the coil wires and connector towards a measurement instrument. This sensor geometry is typical and practically important, but to the best of our knowledge it was not modeled analytically before. The contributions of this paper are the analytical model of the sensor impedance above a plate made of conductive and magnetic material in Section 2 and accompanied Appendix A, analysis of numerical implementation issues in Section 3, comparison with a finite element model in Section 4, experimental verification in Section 5 and sensitivity analysis with respect to the sensor dimension in Section 6. The immediate application of these contributions could allow model-based measurement of material properties and lift-off (e.g., nonconductive coating thickness) with no or less calibration points (measurement above reference standards with known material and lift-off) compared to the existing measurement procedures [26]. The model can also be used in the sensor design phase where it is often necessary to adjust the sensor properties to the specific application or account for sensor production tolerances. Furthermore, we believe that the modeling approach and discussion on the implementation issues can be useful in modeling other similar induction sensors with ferrites.

## 2. Analysis

The geometry of the problem is shown in Figure 1 and Figure 2. The coil with a rectangular cross section is wound around the ferrite core with relative magnetic permeability $\mu_{C}$. The coil and its core are screened with the ferrite shield with relative magnetic permeability $\mu_{S}$. The core and the shield are vertically aligned at the plane z=0. The core and the shield are assumed to be nonconductive. The sensor is located over the magnetic and conductive half space (plate) with electrical conductivity σ and relative magnetic permeability $\mu_{r}$.

Before we derive the vector magnetic potential of the coil, we will first analyze a filamentary sinusoidal current $I\exp\left(j\omega t\right)\delta \left(r-r_{0}\right)\delta \left(z-z_{0}\right)\hat{\varphi }$, where δ is the Dirac delta function and $\hat{\varphi }$ is the azimuthal unit vector for the cylindrical coordinate system. The problem is axially symmetric, so there is only φ component of the vector magnetic potential, i.e., $A\left(r,z\right)=A\left(r,z\right)\hat{\varphi }$.

The problem domain is truncated at far enough boundary r=R, where we will impose the Dirichlet boundary condition $A\left(r=R,z\right)=0$. The consequence of the truncation is that the final expressions for the potential are in series rather than integral form. The problem domain is divided into horizontal regions indicated generally by index *n* in the expressions, or specifically by a number or the letter C. If there are multiple materials or the coil in a region, the region is divided into subregions that are indicated generally by index *m* or specifically by a number following the region designation. For example, $A_{1}$ is the potential in region 1 (no subregions) and $A_{23}$ in region 2, subregion 3.

Following the separation of variables, the general form of the magnetic potential is

(1) $$\begin{array}{cc} \; & A_{n}\left(r,z\right)=\sum_{i=1}^{\infty }R_{n}\left(\alpha_{n,i}r\right)Z_{n}\left(\beta_{n,i}z\right)=\\ \; & =\sum_{i=1}^{\infty }\left[U_{n,i}J_{1}\left(\alpha_{n,i}r\right)+V_{n,i}Y_{1}\left(\alpha_{n,i}r\right)\right]\left[C_{n,i}\exp\left(-\beta_{n,i}z\right)+D_{n,i}\exp\left(\beta_{n,i}z\right)\right],\;\mathrm{for}\;n\in \left\{1,5,6\right\}, \end{array}$$

(2) $$\begin{array}{cc} \; & A_{nm}\left(r,z\right)=\sum_{i=1}^{\infty }R_{nm}\left(\alpha_{n,i}r\right)Z_{n}\left(\alpha_{n,i}z\right)=\\ \; & =\sum_{i=1}^{\infty }\left[U_{nm,i}J_{1}\left(\alpha_{n,i}r\right)+V_{nm,i}Y_{1}\left(\alpha_{n,i}r\right)\right]\left[C_{n,i}\exp\left(-\alpha_{n,i}z\right)+D_{n,i}\exp\left(\alpha_{n,i}z\right)\right],\;\mathrm{for}\;n\in \left\{2,3,4,C\right\}, \end{array}$$

where $J_{1}$ and $Y_{1}$ are first-order Bessel functions of the first and second kind, respectively, $\alpha_{n,i}$ are eigenvalues arising from the Dirichlet boundary condition at r=R for each region, Section 2.2, and

(3) $$\beta_{n,i}=\sqrt{\alpha_{n,i}^{2}+j\omega \mu_{0}\mu_{n}\sigma_{n}}.$$

If a region is homogeneous with relative permeability $\mu_{n}$ and conductivity $\sigma_{n}$, i.e., it has no multiple subregions, the form of the potential is given by (1). For regions with multiple subregions, the general form of the potential in subregion *m* of region *n* with relative permeability $\mu_{nm}$ is given by (2). It is important for further steps to note that there are no conductive materials in the regions with multiple subregions (only air or ferrites) and that, consequently, both *r*-related and *z*-related parts have the same eigenvalues $\alpha_{n,i}$. This means that *z*-related parts of the subregions in a given region are the same in case of (2).

The homogeneous regions $n\in \left\{1,5,6\right\}$ have their corresponding coefficients $V_{i}=0$ because of the divergence of $Y_{1}$ at r=0, so, consequently, one can freely set $U_{i}=1$. Coefficients $U_{nm,i}$ and $V_{nm,i}$ in (2) for regions $n\in \left\{2,3,4,C\right\}$ are determined from the interface conditions along the radial boundaries between the subregions, as described in Section 2.1. The interface conditions in this case are continuity of $B_{r}$ and $H_{z}$, i.e., component of B in $\hat{r}$ direction, and component of H in $\hat{z}$ direction.

The unknown coefficients $C_{n,i}$ and $D_{n,i}$ in (1) and (2) are determined from the boundary and interface conditions along the horizontal boundaries between the regions, Section 2.4. The potential must remain finite as z→±∞, so $D_{1,i}=0$ and $C_{6,i}=0$. The interface conditions for the horizontal boundaries are continuity of $B_{z}$ and $H_{r}$.

### 2.1. Continuity of $B_{r}$ and $H_{z}$ at Radial Boundaries between Subregions

This condition has to be satisfied for regions 2, 3 and 4. We will analyze a general case of a region *n* with subregions m=1,…,M and radial interfaces between these subregions at $r_{n1},\ldots ,r_{n(M-1)}$. In that case, the radial parts under the sum in (2) for each subregion are

(4) $$\begin{array}{c} R_{n1}\left(\alpha_{n,i}r\right)=J_{1}\left(\alpha_{n,i}r\right),\;0\leq r\leq r_{n1}, \end{array}$$

(5) $$\begin{array}{c} R_{n2}\left(\alpha_{n,i}r\right)=U_{n2,i}J_{1}\left(\alpha_{n,i}r\right)+V_{n2,i}Y_{1}\left(\alpha_{n,i}r\right),\;r_{n1}<r\leq r_{n2},\\ \vdots \end{array}$$

(6) $$\begin{array}{c} R_{nm}\left(\alpha_{n,i}r\right)=U_{nm,i}J_{1}\left(\alpha_{n,i}r\right)+V_{nm,i}Y_{1}\left(\alpha_{n,i}r\right),\;r_{n(m-1)}<r\leq r_{nm},\\ \vdots \end{array}$$

(7) $$\begin{array}{c} R_{nM}\left(\alpha_{n,i}r\right)=U_{nM,i}J_{1}\left(\alpha_{n,i}r\right)+V_{nM,i}Y_{1}\left(\alpha_{n,i}r\right),\;r_{n(M-1)}<r\leq R,\;r_{nM}=R. \end{array}$$

For the two neighboring subregions *m* and m+1, the radial interface of continuity of $B_{r}$ and $H_{z}$ at $r=r_{nm}$ is equivalent to

(8) $$\begin{array}{c} A_{nm}\left(r_{nm},z\right)=A_{n(m+1)}\left(r_{nm},z\right), \end{array}$$

(9) $$\begin{array}{c} \frac{1}{\mu_{nm}}\left(\frac{A_{nm}}{r}+\frac{\partial A_{nm}}{\partial r}\right){|}_{r=r_{nm}}=\frac{1}{\mu_{n(m+1)}}\left(\frac{A_{n(m+1)}}{r}+\frac{\partial A_{n(m+1)}}{\partial r}\right){|}_{r=r_{nm}}. \end{array}$$

Since all subregions in region *n* have the same *z*-related part, the radial interface conditions can be satisfied by adjusting the coefficients of the *r*-related part only. The notation will be simpler if we define matrix R as

(10) $$R\left(\alpha_{n,i}r_{nm},\mu_{nm}\right)=\left[\begin{array}{cc} J_{1}\left(\alpha_{n,i}r_{nm}\right) & Y_{1}\left(\alpha_{n,i}r_{nm}\right)\\ J_{0}\left(\alpha_{n,i}r_{nm}\right)/\mu_{nm} & Y_{0}\left(\alpha_{n,i}r_{nm}\right)/\mu_{nm} \end{array}\right].$$

It can be shown using (2)–(10) that the coefficients in the *r*-related part of subregion m+1 are connected to the coefficients of subregion *m* as

(11) $$\left[\begin{array}{c} U_{n(m+1),i}\\ V_{n(m+1),i} \end{array}\right]=R^{-1}\left(\alpha_{n,i}r_{nm},\mu_{n(m+1)}\right)R\left(\alpha_{n,i}r_{nm},\mu_{nm}\right)\left[\begin{array}{c} U_{nm,i}\\ V_{nm,i} \end{array}\right].$$

Using recurrence relation (11), and since $V_{n1,i}=0$ and $U_{n1,i}$ can be freely set to 1, we can determine the *r*-related coefficients for all subregions in a given region:

(12) $$\left[\begin{array}{c} U_{n1,i}\\ V_{n1,i} \end{array}\right]=\left[\begin{array}{c} 1\\ 0 \end{array}\right],\;\mathrm{for}\;m=1,\;0\leq r\leq r_{n1},$$

(13) $$\left[\begin{array}{c} U_{n2,i}\\ V_{n2,i} \end{array}\right]=R^{-1}\left(\alpha_{n,i}r_{n1},\mu_{n2}\right)\left[\begin{array}{c} J_{1}\left(\alpha_{n,i}r_{n1}\right)\\ J_{0}\left(\alpha_{n,i}r_{n1}\right)/\mu_{n1} \end{array}\right],\;\mathrm{for}\;m=2,\;r_{n1}<r\leq r_{n2},$$

(14) $$\left[\begin{array}{c} U_{nm,i}\\ V_{nm,i} \end{array}\right]=\prod_{k=3}^{m}\left(R^{-1}\left(\alpha_{n,i}r_{n(k-1)},\mu_{nk}\right)R\left(\alpha_{n,i}r_{n(k-1)},\mu_{n(k-1)}\right)\right)\left[\begin{array}{c} U_{n2,i}\\ V_{n2,i} \end{array}\right],\;\begin{array}{c} \mathrm{for}\;m=3,\ldots ,M,\;r_{n(m-1)}<r\leq r_{nm},\;r_{nM}=R. \end{array}$$

Because coefficients $U_{nm,i}$ and $V_{nm,i}$ do not depend on spatial coordinates *r* and *z*, the radial parts $R_{nm}\left(\alpha_{n,i}r\right)$ given in (4)–(7) are linear combinations of Bessel functions $J_{1}$ and $Y_{1}$ with respect to *r*. This will be important later on when we make use of the orthogonality property of the Bessel functions. In order to facilitate this, we will add an index to $R_{nm}$ to indicate the order of the involved Bessel functions:

(15) $$R_{\nu ,nm}\left(\alpha_{n,i}r\right)=U_{nm,i}J_{\nu }\left(\alpha_{n,i}r\right)+V_{nm,i}Y_{\nu }\left(\alpha_{n,i}r\right),$$

where coefficients $U_{nm,i}$ and $V_{nm,i}$ do not depend on ν. It follows from (8) and (9) that

(16) $$\begin{array}{c} R_{1,nm}\left(\alpha_{n,i}r_{nm}\right)=R_{1,n\left(m+1\right)}\left(\alpha_{n,i}r_{nm}\right), \end{array}$$

(17) $$\begin{array}{c} \frac{1}{\mu_{nm}}R_{0,nm}\left(\alpha_{n,i}r_{nm}\right)=\frac{1}{\mu_{n(m+1)}}R_{0,n\left(m+1\right)}\left(\alpha_{n,i}r_{nm}\right). \end{array}$$

### 2.2. Eigenvalues

The radial parts of all regions and subregions are defined in the preceding section. The first step in their computation is to find the eigenvalues that satisfy the Dirichlet condition at the truncated boundary of the problem:

(18) $$\begin{array}{c} A_{n}\left(r=R,z\right)=0\Rightarrow J_{1}\left(\alpha_{n,i}R\right)=0,\;\mathrm{for}\;\mathrm{homogeneous}\;\mathrm{region}, \end{array}$$

(19) $$\begin{array}{c} A_{nM}\left(r=R,z\right)=0\Rightarrow R_{1,nM}\left(\alpha_{n,i}R\right)=0,\;\mathrm{for}\;\mathrm{region}\;\mathrm{with}\;M\;\mathrm{subregions}. \end{array}$$

The eigenvalues $\alpha_{n,i}$ are the real and positive roots of (18) and (19). Homogeneous regions 1, 5 and 6 have identical radial parts and, hence, the same eigenvalues. Similarly, the regions 3, 4 and C, with multiple subregions, have the same radial parts and the same eigenvalues. Region 2 has its own eigenvalues. In order to simplify the notation, which was so far useful for the general case, we will designate the elements of the three sets of eigenvalues as $\alpha_{i}$, $p_{i}$ and $q_{i}$:

(20) $$\begin{array}{c} \alpha_{i}=\alpha_{1,i}=\alpha_{5,i}=\alpha_{6,i}, \end{array}$$

(21) $$\begin{array}{c} p_{i}=\alpha_{2,i}, \end{array}$$

(22) $$\begin{array}{c} q_{i}=\alpha_{3,i}=\alpha_{4,i}=\alpha_{C,i}, \end{array}$$

and for the *z*-related part in region 6

(23) $$\beta_{i}=\beta_{6,i}=\sqrt{\alpha_{i}^{2}+j\omega \mu_{0}\mu_{r}\sigma }.$$

### 2.3. Final Expressions for Potential in All Regions in Matrix Form

Infinite series in (1) and (2) have to be truncated to *N* terms in the computation. The potential is

(24) $$A_{1}\left(r,z\right)=\sum_{i=1}^{N}J_{1}\left(\alpha_{i}r\right)\exp\left(-\alpha_{i}z\right)C_{1,i}/\alpha_{i},$$

(25) $$A_{2}\left(r,z\right)=\sum_{i=1}^{N}\left\{\begin{array}{cc} J_{1}\left(p_{i}r\right), & \mathrm{if}\;0\leq r\leq r_{S1}\\ R_{1,22}\left(p_{i}r\right), & \mathrm{if}\;r_{S1}<r\leq r_{S2}\\ R_{1,23}\left(p_{i}r\right), & \mathrm{if}\;r_{S2}<r\leq R \end{array}\right\}\left[\exp\left(-p_{i}z\right)C_{2,i}+\exp\left(p_{i}z\right)D_{2,i}\right]/p_{i},$$

(26) $$A_{3}\left(r,z\right)=\sum_{i=1}^{N}\left\{\begin{array}{cc} J_{1}\left(q_{i}r\right), & \mathrm{if}\;0\leq r\leq r_{C}\\ R_{1,32}\left(q_{i}r\right), & \mathrm{if}\;r_{C}<r\leq r_{S1}\\ R_{1,33}\left(q_{i}r\right), & \mathrm{if}\;r_{S1}<r\leq r_{S2}\\ R_{1,34}\left(q_{i}r\right), & \mathrm{if}\;r_{S2}<r\leq R \end{array}\right\}\left[\exp\left(-q_{i}z\right)C_{3,i}+\exp\left(q_{i}z\right)D_{3,i}\right]/q_{i},$$

(27) $$A_{4}\left(r,z\right)=\sum_{i=1}^{N}\left\{\begin{array}{cc} J_{1}\left(q_{i}r\right), & \mathrm{if}\;0\leq r\leq r_{C}\\ R_{1,42}\left(q_{i}r\right), & \mathrm{if}\;r_{C}<r\leq r_{S1}\\ R_{1,43}\left(q_{i}r\right), & \mathrm{if}\;r_{S1}<r\leq r_{S2}\\ R_{1,44}\left(q_{i}r\right), & \mathrm{if}\;r_{S2}<r\leq R \end{array}\right\}\left[\exp\left(-q_{i}z\right)C_{4,i}+\exp\left(q_{i}z\right)D_{4,i}\right]/q_{i},$$

(28) $$A_{5}\left(r,z\right)=\sum_{i=1}^{N}J_{1}\left(\alpha_{i}r\right)\left[\exp\left(-\alpha_{i}z\right)C_{5,i}+\exp\left(\alpha_{i}z\right)D_{5,i}\right]/\alpha_{i},$$

(29) $$A_{5}\left(r,z\right)=\sum_{i=1}^{N}J_{1}\left(\alpha_{i}r\right)\left[\exp\left(-\alpha_{i}z\right)C_{5,i}+\exp\left(\alpha_{i}z\right)D_{5,i}\right]/\alpha_{i},$$

(30) $$A_{6}\left(r,z\right)=\sum_{i=1}^{N}J_{1}\left(\alpha_{i}r\right)\exp\left(\beta_{i}z\right)D_{6,i}/\beta_{i}.$$

In the above expressions, we divided with the corresponding eigenvalues in order to make later expressions somewhat simpler, which is allowed because it will be canceled out by the final form of $C_{n,i}$ and $D_{n,i}$ [17].

It is convenient to write (24)–(30) in the matrix form. To do that, we will assume that the functions $J_{\nu }\left(\right)$, $R_{\nu ,nm}\left(\right)$, exp(), as well as the integration and differentiation are applied element wise on a vector or matrix. We will use boldface type to represent matrices (e.g., α or $\exp\left(qz\right)$), overlined italic type to represent row vectors (e.g., $\bar{\alpha }$ or $J_{1}\left(\bar{\alpha }r\right)$) and underlined italic type to represent column vectors (e.g., ${\underline{C}}_{1}$). The dimensions of the matrices are N×N, row vectors 1×N and column vectors N×1. This notation eases implementation in a programming language such as Matlab or Julia. Using this notation, the matrix form of (24)–(30) is

(31) $$\begin{array}{c} A_{1}\left(r,z\right)=J_{1}\left(\bar{\alpha }r\right)\alpha^{-1}\exp\left(-\alpha z\right){\underline{C}}_{1}, \end{array}$$

(32) $$\begin{array}{c} A_{2}\left(r,z\right)=\left\{\begin{array}{c} J_{1}\left(\bar{p}r\right)\\ R_{1,22}\left(\bar{p}r\right)\\ R_{1,23}\left(\bar{p}r\right) \end{array}\right\}p^{-1}\left[\exp\left(-pz\right){\underline{C}}_{2}+\exp\left(pz\right){\underline{D}}_{2}\right], \end{array}$$

(33) $$\begin{array}{c} A_{3}\left(r,z\right)=\left\{\begin{array}{c} J_{1}\left(\bar{q}r\right)\\ R_{1,32}\left(\bar{q}r\right)\\ R_{1,33}\left(\bar{q}r\right)\\ R_{1,34}\left(\bar{q}r\right) \end{array}\right\}q^{-1}\left[\exp\left(-qz\right){\underline{C}}_{3}+\exp\left(qz\right){\underline{D}}_{3}\right], \end{array}$$

(34) $$\begin{array}{c} A_{4}\left(r,z\right)=\left\{\begin{array}{c} J_{1}\left(\bar{q}r\right)\\ R_{1,42}\left(\bar{q}r\right)\\ R_{1,43}\left(\bar{q}r\right)\\ R_{1,44}\left(\bar{q}r\right) \end{array}\right\}q^{-1}\left[\exp\left(-qz\right){\underline{C}}_{4}+\exp\left(qz\right){\underline{D}}_{4}\right], \end{array}$$

(35) $$\begin{array}{c} A_{5}\left(r,z\right)=J_{1}\left(\bar{\alpha }r\right)\alpha^{-1}\left[\exp\left(-\alpha z\right){\underline{C}}_{5}+\exp\left(\alpha z\right){\underline{D}}_{5}\right], \end{array}$$

(36) $$\begin{array}{c} A_{6}\left(r,z\right)=J_{1}\left(\bar{\alpha }r\right)\beta^{-1}\exp\left(\beta z\right){\underline{D}}_{6}. \end{array}$$

In the above expressions, matrices α, p and q are diagonal with elements of $\bar{\alpha }$, $\bar{p}$ and $\bar{q}$ on their main diagonal, respectively.

### 2.4. Continuity of $B_{z}$ and $H_{r}$ for Horizontal Boundaries between Regions

The continuity of $B_{z}$ and $H_{r}$ have to be satisfied along the horizontal interface (z=const.) between two regions. For two neighboring regions *n* and n+1, the horizontal interface condition at $z=z_{n}$ is equivalent to

(37) $$\begin{array}{c} \left(\frac{A_{n+1}}{r}+\frac{\partial A_{n+1}}{\partial r}\right){|}_{z=z_{n}}-\left(\frac{A_{n}}{r}+\frac{\partial A_{n}}{\partial r}\right){|}_{z=z_{n}}=0, \end{array}$$

(38) $$\begin{array}{c} \frac{1}{\mu_{n+1}}\frac{\partial A_{n+1}}{\partial z}{|}_{z=z_{n}}-\frac{1}{\mu_{n}}\frac{\partial A_{n}}{\partial z}{|}_{z=z_{n}}=\left\{\begin{array}{cc} \mu_{0}I\delta \left(r-r_{0}\right), & \mathrm{if}\;z_{n}=z_{0},\\ 0, & \mathrm{otherwise}. \end{array}\right. \end{array}$$

Relative permeability $\mu_{n}$ in (38) is the piecewise constant function of *r* for region *n* if it has multiple subregions, i.e., $\mu_{n}=\mu_{n1}$ for $0\leq r\leq r_{n1}$,…, $\mu_{n}=\mu_{nM}$ for $r_{n(M-1)}<r\leq R$.

The interface relations that have to be satisfied for all five horizontal boundaries ($z=L_{S}$, $z=L_{C}$, $z=z_{0}$, z=0 and z=−h) are given in Appendix A for the sake of clarity. In order for these relations, e.g., (A3), to be satisfied for the entire interval 0≤r≤R, the procedure followed in Appendix A is to expand the *r*-dependent parts using a basis formed of continuous piecewise linear combinations of Bessel functions of the first and second kind. The choice of the basis is somewhat arbitrary but it is better, from the standpoint of numerical implementation, to choose these in such a way that as many of the resulting matrices are identical or diagonal. Term-by-term comparison results in a matrix equation for each interface relation involving corresponding vectors $\underline{C}$ and $\underline{D}$. This procedure has to be repeated twice (continuity of $B_{z}$ and $H_{r}$) for each of the 5 horizontal interfaces giving rise to 10 algebraic equations with matrix coefficients and 10 unknown vectors $\underline{C}$ and $\underline{D}$.

### 2.5. Calculation of Coefficients $\underline{C}$ and $\underline{D}$

Coefficient vectors $\underline{C}$ and $\underline{D}$ in (31)–(36) for each of the regions are calculated by solving 10×10 system of algebraic equations derived in Appendix A, i.e., (A5), (A10), (A13), (A16), (A18), (A21), (A25), (A28), (A30) and (A31). First, we define some auxiliary expressions sorted in order of their computational dependencies:

(39) $$\begin{array}{c} T_{1}=\exp\left[-p\left(L_{S}-L_{C}\right)\right]\left[2E{\left(E+F\right)}^{-1}-I\right]\exp\left[-p\left(L_{S}-L_{C}\right)\right], \end{array}$$

(40) $$\begin{array}{c} \Lambda =\frac{\mu_{r}\alpha -\beta }{\mu_{r}\alpha +\beta }\exp\left(-2\alpha h\right), \end{array}$$

(41) $$\begin{array}{c} W^{\prime }=\left(I-T_{1}\right)W, \end{array}$$

(42) $$\begin{array}{c} L^{\prime }=\left(I+T_{1}\right)L, \end{array}$$

(43) $$\begin{array}{c} V^{\prime }=V\left(I+\Lambda \right), \end{array}$$

(44) $$\begin{array}{c} U^{\prime }=U\left(I-\Lambda \right), \end{array}$$

(45) $$\begin{array}{c} T_{2}=-\exp\left(-qL_{C}\right){\left(W^{\prime }+L^{\prime }\right)}^{-1}\left(W^{\prime }-L^{\prime }\right)\exp\left(-qL_{C}\right), \end{array}$$

(46) $$\begin{array}{c} T_{3}=\frac{1}{2}\left[\left(I-T_{2}\right)S^{-1}V^{\prime }+\left(I+T_{2}\right)S^{-1}U^{\prime }\right], \end{array}$$

(47) $$\begin{array}{c} T_{4}=\left[W+L-\left(W-L\right){\left(W^{\prime }+L^{\prime }\right)}^{-1}\left(W^{\prime }-L^{\prime }\right)\right]\exp\left(-qL_{C}\right). \end{array}$$

For the filamentary coil at $\left(r_{0},z_{0}\right)$:

(48) $$\begin{array}{c} \underline{X}=\frac{1}{2}\mu_{0}IS^{-1}r_{0}R_{1,32}\left(\underline{q}r_{0}\right), \end{array}$$

(49) $$\begin{array}{c} X_{\mathrm{pos}}=\exp\left(qz_{0}\right), \end{array}$$

(50) $$\begin{array}{c} X_{\mathrm{neg}}=\exp\left(-qz_{0}\right). \end{array}$$

For the coil with the rectangular cross section:

(51) $$\begin{array}{c} \underline{X}=\frac{1}{2}\mu_{0}i_{\mathrm{TX}}S^{-1}q^{-3}\mathrm{int}\left(\underline{q}r_{T1},\underline{q}r_{T2}\right), \end{array}$$

(52) $$\begin{array}{c} X_{\mathrm{pos}}=\exp\left(qz_{T2}\right)-\exp\left(qz_{T1}\right), \end{array}$$

(53) $$\begin{array}{c} X_{\mathrm{neg}}=-\exp\left(-qz_{T2}\right)+\exp\left(qz_{T1}\right), \end{array}$$

where

(54) $$\mathrm{int}\left(x_{1},x_{2}\right)=\int_{x_{1}}^{x_{2}}xR_{1,32}\left(x\right)dx.$$

Finally, we can write the coefficients $\underline{C}$ and $\underline{D}$ sorted in order of their computational dependencies:

(55) $$\begin{array}{c} {\underline{D}}_{5}=T_{3}^{-1}\left(T_{2}X_{\mathrm{pos}}+X_{\mathrm{neg}}\right)\underline{X}, \end{array}$$

(56) $$\begin{array}{c} {\underline{C}}_{5}=\Lambda {\underline{D}}_{5}, \end{array}$$

(57) $$\begin{array}{c} {\underline{D}}_{6}=2\frac{\mu_{r}\beta }{\mu_{r}\alpha +\beta }\exp\left[\left(\beta -\alpha \right)h\right]{\underline{D}}_{5}, \end{array}$$

(58) $$\begin{array}{c} {\underline{C}}_{4}=\frac{1}{2}S^{-1}\left(V^{\prime }-U^{\prime }\right){\underline{D}}_{5}, \end{array}$$

(59) $$\begin{array}{c} {\underline{D}}_{4}=\frac{1}{2}S^{-1}\left(V^{\prime }+U^{\prime }\right){\underline{D}}_{5}, \end{array}$$

(60) $$\begin{array}{c} {\underline{C}}_{3}={\underline{C}}_{4}+X_{\mathrm{pos}}\underline{X}, \end{array}$$

(61) $$\begin{array}{c} {\underline{D}}_{3}={\underline{D}}_{4}-X_{\mathrm{neg}}\underline{X}, \end{array}$$

(62) $$\begin{array}{c} {\underline{C}}_{2}=\frac{1}{2}\exp\left(pL_{C}\right)K^{-1}T_{4}{\underline{C}}_{3}, \end{array}$$

(63) $$\begin{array}{c} {\underline{D}}_{2}=\frac{1}{2}\exp\left(-pL_{S}\right)K^{-1}\left[2E{\left(E+F\right)}^{-1}-I\right]\exp\left[-p\left(L_{S}-L_{C}\right)\right]T_{4}{\underline{C}}_{3}, \end{array}$$

(64) $$\begin{array}{c} {\underline{C}}_{1}=\exp\left(\alpha L_{S}\right){\left(E+F\right)}^{-1}\exp\left[-p\left(L_{S}-L_{C}\right)\right]T_{4}{\underline{C}}_{3}. \end{array}$$

### 2.6. Coil with Rectangular Cross Section

The magnetic potential of a coil with rectangular cross section and $N_{\mathrm{TX}}$ turns, carrying sinusoidal current of amplitude $I_{\mathrm{TX}}$, is calculated by the superposition of a number of filamentary coils. Let a coil with infinitesimal cross section $dr_{0}dz_{0}$ carrying current $i_{\mathrm{TX}}dr_{0}dz_{0}$ approach a filamentary current *I* at $\left(r_{0},z_{0}\right)$, where the current density $i_{\mathrm{TX}}$ is

(65) $$i_{\mathrm{TX}}=\frac{N_{\mathrm{TX}}I_{\mathrm{TX}}}{\left(r_{T2}-r_{T1}\right)\left(z_{T2}-z_{T1}\right)}.$$

In that case, the total magnetic potential for region *n* is

(66) $$A_{n}^{\mathrm{tot}}\left(r,z\right)=\int_{r_{T1}}^{r_{T2}}\int_{z_{T1}}^{z_{T2}}A_{n}\left(r,z,r_{0},z_{0}\right)dr_{0}dz_{0},$$

where $A_{n}\left(r,z,r_{0},z_{0}\right)$ is given by (31)–(36) depending on the region. Integration in (66) comes down to integration of $X_{\mathrm{pos}}\underline{X}$ and $X_{\mathrm{neg}}\underline{X}$ in (48)–(50), and it results in (51)–(53). The total potential $A_{n}^{\mathrm{tot}}$ is given then by the same expressions as $A_{n}$ in (31)–(36), except one uses (51)–(53) instead of (48)–(50) in (55), and (60) and (61) for ${\underline{D}}_{5}$, ${\underline{C}}_{3}$ and ${\underline{D}}_{3}$, respectively. One should note that superposition for region C ($z_{T1}\leq z\leq z_{T1}$), which is present only in the case of the rectangular cross section coil, requires integration of $A_{3}\left(r,z,r_{0},z_{0}\right)$ for $z_{T1}\leq z_{0}\leq z$ and $A_{4}\left(r,z,r_{0},z_{0}\right)$ for $z\leq z_{0}\leq z_{T2}$, i.e.,

(67) $$A_{C}\left(r,z\right)=\int_{r_{T1}}^{r_{T2}}\int_{z_{T1}}^{z}A_{3}\left(r,z,r_{0},z_{0}\right)dr_{0}dz_{0}+\int_{r_{T1}}^{r_{T2}}\int_{z}^{z_{T2}}A_{4}\left(r,z,r_{0},z_{0}\right)dr_{0}dz_{0}.$$

It can be shown that (67) results in

(68) $$\begin{array}{c} A_{C}\left(r,z\right)=A_{4}^{\mathrm{tot}}\left(r,z\right)+\\ \;\;\;\;+\left\{\begin{array}{c} J_{1}\left(\bar{q}r\right)\\ R_{1,42}\left(\bar{q}r\right)\\ R_{1,43}\left(\bar{q}r\right)\\ R_{1,44}\left(\bar{q}r\right) \end{array}\right\}q^{-1}\left[2I-\exp\left(-qz\right)\exp\left(qz_{T1}\right)-\exp\left(qz\right)\exp\left(-qz_{T1}\right)\right]\underline{X}, \end{array}$$

where $\underline{X}$ is given by (51). The impedance of the coil, which is the most important output of the model from the measurement point of view, is calculated by the integration of the voltage induced in a single loop over the coil cross section:

(69) $$Z=j\omega \frac{2\pi N_{\mathrm{TX}}}{\left(r_{T2}-r_{T1}\right)\left(z_{T2}-z_{T1}\right)I_{TX}}\int_{r_{T1}}^{r_{T2}}\int_{z_{T1}}^{z_{T2}}rA_{C2}\left(r,z\right)drdz,$$

where $A_{C2}$ is the potential in region C, subregion 2. Finally, the impedance of the coil is

(70) $$\begin{array}{c} Z=j\omega \frac{2\pi N_{\mathrm{TX}}}{\left(r_{T2}-r_{T1}\right)\left(z_{T2}-z_{T1}\right)I_{TX}}\mathrm{int}\left(\bar{q}r_{T1},\bar{q}r_{T2}\right)q^{-4}\\ \;\;\;[-\left(\exp\left(-qz_{T2}\right)-\exp\left(-qz_{T1}\right)\right){\underline{C}}_{4}+\left(\exp\left(qz_{T2}\right)-\exp\left(qz_{T1}\right)\right){\underline{D}}_{4}+\\ \;\;\;\;+\left(2q\left(z_{T2}-z_{T1}\right)+\exp\left[-q\left(z_{T2}-z_{T1}\right)\right]-\exp\left[q\left(z_{T2}-z_{T1}\right)\right]\right)\underline{X}]. \end{array}$$

The voltage induced in a receiver coil can be calculated using an approach analogous to (69) with the choice of the potential corresponding to the location of the receiver coil.

## 3. Numerical Implementation

The model can be implemented in a programming language suitable for numerical analysis, such as Matlab or Julia. We have chosen the former. The major functional parts of the implementation are:

1. Calculation of coefficients for the radial part $R_{nm}\left(\alpha_{n,i}r\right)$ given in (4)–(7) using (12)–(14).
2. Root finding of (18) and (19) in order to obtain the required eigenvalues.
3. Integration of $\mathrm{int}\left(x_{1},x_{2}\right)$ in (54).
4. Calculation of the matrices in Appendix A (E, K, F, W, L, U, V and S) to form the set of 10 algebraic equations for 10 unknown coefficients $\underline{C}$ and $\underline{D}$.
5. Calculation of the coefficients $\underline{C}$ and $\underline{D}$ using equations in Section 2.5.
6. Calculation of the coil impedance *Z* using (70).

While steps 1, 4 and 6 were implemented straightforwardly following the relevant expressions in the paper, the remaining ones deserve some additional comments.

The functions on the left side of Equations (18) and (19) are real and oscillatory. We found their roots by sampling the functions so that intervals containing exactly one zero crossing can be selected and the roots bracketed. As a root finding algorithm, we used Anderson–Björck modification of the classic regula falsi method [27].

In general, the form of the integral in (54) involving linear combination of Bessel functions of the first and second kind, $J_{1}$ and $Y_{1}$, is more easily calculated using Struve functions or expansion in Chebyshev polynomials than by direct numerical integration [18]. We used Struve functions $H_{0}$ and $H_{1}$ calculated via their expansion into the series of Bessel functions for which the relevant identities can be found in [28]:

(71) $$\int_{x_{1}}^{x_{2}}tR_{1,nm}\left(t\right)dt=\frac{\pi t}{2}\left[H_{0}\left(t\right)R_{1,nm}\left(t\right)-H_{1}\left(t\right)R_{0,nm}\left(t\right)\right]{|}_{x_{1}}^{x_{2}}.$$

The expressions for $\underline{C}$ and $\underline{D}$ in Section 2.5 require some matrices to be inverted. Increasing the length *N* of the series representation increases the eigenvalues. The factors containing exponential functions with positively signed products of an eigenvalue and a spatial dimension can cause a loss of precision and a poor condition number of the matrices that need to be inverted. Thus, care should be taken to arrange the final expressions in such a way that as much as possible of the matrices are diagonal and that the exponential functions have arguments with negative sign.

## 4. Comparison with FEM

In order to verify the model of the sensor impedance, we compared the model predictions with the results of a finite elements model (FEM) study. In both numerical studies we used the sensor with properties as in Table 1. For the FEM study we used free, simple and thoroughly verified 2D solver Finite Element Model Magnetics (FEMM) that was successfully used in a number of studies [29]. The number of the series terms in the presented model of the sensor impedance was N=140 and the domain boundary was set to $R=10r_{S2}$. The identical geometry in FEMM was meshed with approximately 2.4 million of triangular elements. The computation time for one realization of the problem (i.e., for the selected dimensions, frequency and properties of the core, shield and medium) was around 30 min for FEMM, and less than 0.7 s for the calculation of the model, including the eigenvalues computation and without usage of precalculated values.

**Figure 3:**
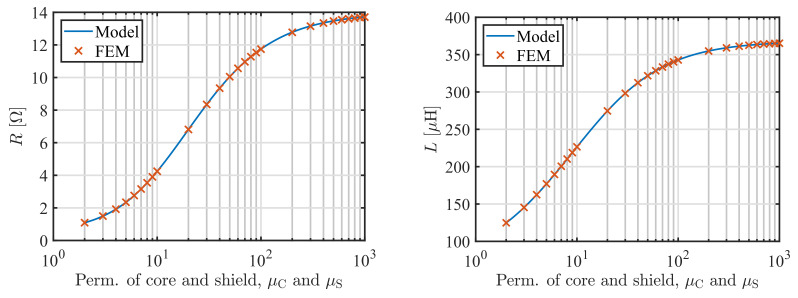
Comparison of the model predictions and results of the FEM study for resistance *R* (**left**) and inductance *L* (**right**) of the sensor depending on the permeability of the core $\mu_{C}$ and shield $\mu_{S}$ assuming that $\mu_{C}=\mu_{S}$. The plate has σ=5 MS/m and $\mu_{r}=50$, and the frequency is 60 kHz.

First, we calculated the impedance of the coil depending on the permeability of the core and shield for the plate with σ=5 MS/m and $\mu_{r}=50$ at 60 kHz, Figure 3. For the sake of simplicity, we assumed that $\mu_{C}=\mu_{S}$. There is practically no significant further change in the impedance of the sensor for the permeability above 500, which is a finding observed for other cored coils as well [17]. The relative discrepancy between the model and FEM results are bellow 0.07% for the inductance and 0.23% for the resistance. These discrepancies can be further reduced by increasing the density of the FEM mesh.

**Figure 4:**
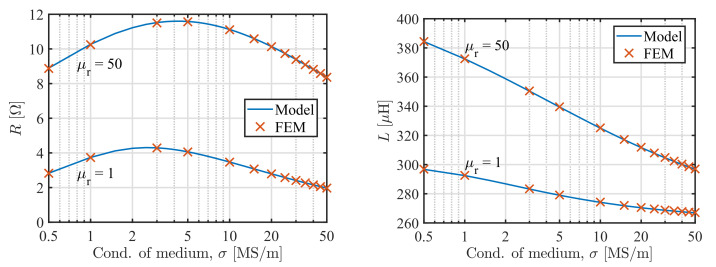
Comparison of the model predictions and results of the FEM study for resistance *R* (**left**) and inductance *L* (**right**) of the sensor at 60 kHz depending on the conductivity of the medium σ for two cases of the permeability of the plate $\mu_{r}=1$ and $\mu_{r}=50$. The core has $\mu_{C}=100$ and the shield $\mu_{S}=50$.

Second, we calculated the impedance of the coil depending on the conductivity σ of the plate for two values of its permeability $\mu_{r}=1$ and $\mu_{r}=50$ at 60 kHz, Figure 4. The core permeability $\mu_{C}=100$ and the shield permeability $\mu_{S}=50$. Typical dependency of the coil impedance on the plate conductivity can be observed, including the maximum point of the resistance [17]. The relative discrepancy between the model and FEM results are bellow 0.07% for the inductance and 0.25% for the resistance.

**Figure 5:**
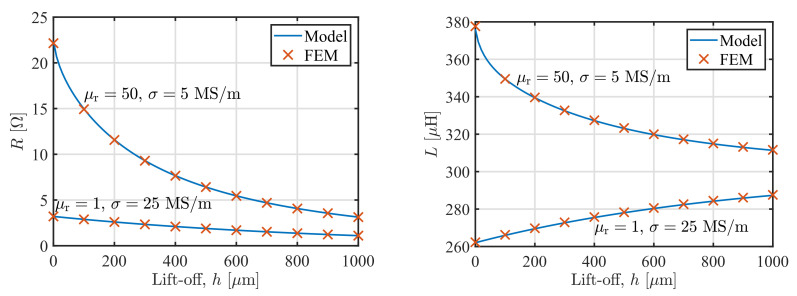
Comparison of the model predictions and results of the FEM study for resistance *R* (**left**) and inductance *L* (**right**) of the sensor at 60 kHz depending on the lift-off *h* for two plates: $\mu_{r}=1$ and σ=25 MS/m, and $\mu_{r}=50$ and σ=5 MS/m. The core has $\mu_{C}=100$ and the shield $\mu_{S}=50$.

Finally, we calculated the impedance of the coil depending on the lift-off *h* of the sensor placed above either a nonmagnetic plate with $\mu_{r}=1$ and σ=25 MS/m (properties similar to an aluminum alloy), and a magnetic plate $\mu_{r}=50$ and σ=5MS/m (properties similar to a carbon steel), Figure 5. The core permeability was $\mu_{C}=100$ and the shield permeability $\mu_{S}=50$. Again, the relative discrepancy between the model and FEM results are bellow 0.07% for the inductance and 0.25% for the resistance. As expected, the resistance decreases with the lift-off increase for both plates, whereas the inductance in the case of the nonmagnetic plate increases with the lift-off, and decreases in the case of the magnetic plate.

## 5. Comparison with Experimental Results

We manufactured the sensor according to the geometry in Figure 1 and Figure 2. The sensor properties are given in Table 2. The impedance of the sensor in the air and above an aluminum plate was measured using the precision LCR meter HP 4284A in the frequency range from 5 kHz to 100 kHz and for lift-off values from 0 μm to 1000 μm in 100 μm steps. The plate was made of aluminium alloy EN AW-6082 with a conductivity of 42.2%IACS or 24.56 MS/m. The impedance of the coil *Z* is the sum of the coil impedance in air (without the plate) $Z_{0}=j\omega L_{0}$, and the impedance change ΔZ=ΔR+jωΔL due to the proximity of the plate:

(72) $$Z=j\omega L_{0}+\Delta Z,$$

where the resistance of the coil in air and the coil parasitic capacitance have been compensated for.

**Figure 6:**
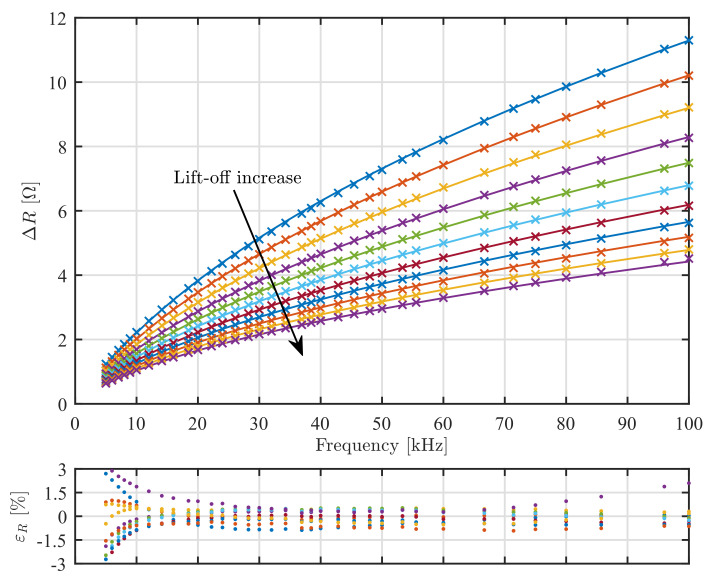
Real part ΔR of impedance change ΔZ. Full lines are modeling results and crosses are measured values. The top curve is for lift-off 0 μm and the bottom curve for 1000 μm. Other curves are in 100 μm increments. The graph bellow shows error $\varepsilon_{R}$ of individual measurement data points relative to the model prediction.

**Figure 7:**
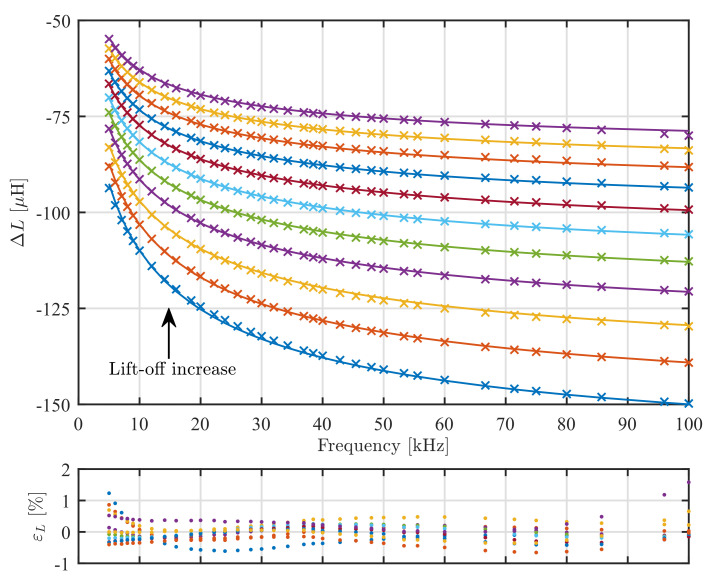
Imaginary part ΔL of impedance change ΔZ. Full lines are modeling results and crosses are measured values. The bottom curve is for lift-off 0 μm and the top curve for 1000 μm. Other curves are in 100 μm increments. The graph bellow shows error $\varepsilon_{L}$ of individual measurement data points relative to the model prediction.

The inductance of the sensor in the air was calculated 1525.67 μH, while the measured value was from 1524.48 μH to 1526.38 μH depending on the frequency. The impedance change obtained from the model and the experiment are shown in Figure 6 and Figure 7 for ΔR and ΔL, respectively. The model and experiment are in practically acceptable agreement with the relative error of ΔR within ±3% and ΔL within ±1%. These errors are comparable to the ones in other studies on eddy current sensor modeling. In practice, model-based measurements of conductivity and lift-off require calibration procedures with samples of known conductivity and lift-off if one wants to achieve measurement uncertainty better than the reported modeling errors would allow.

## 6. Sensitivity to Sensor Dimensions

The data in Table 2 was obtained by averaging and rounding to 0.01 mm the values measured using a Mitutoyo micrometer with resolution of 0.001 mm and accuracy of ±2 μm. We rounded the values because at a higher resolution, the measurement is affected by the applied pressure or measurement position along the measured object, e.g., the ferrite core was not a perfect cylinder at a resolution of 0.001 mm. We used purposefully overestimated measurement uncertainty of ±0.01 mm to evaluate its effect on the sensor impedance. With that aim, we calculated the sensitivity of the sensor impedance to the sensor dimensions. Table 3 shows the relative changes of the inductance in the air $L_{0}$ and impedance change ΔR and ΔL (for plate with σ=5 MS/m and $\mu_{r}=50$ and lift-off 0 μm) if each of the nine sensor dimensions is independently varied within the interval of ±0.01 mm. The core radius and the shield inner radius uncertainties have the most significant contribution to the impedance uncertainty. This means that the gap between the core and the shield must be precisely controlled during the sensor production.

## 7. Conclusions

The validity of the impedance model is corroborated by a practically negligible discrepancy in comparison with the FEM approach. We compared the model and FEM approach for a range of practical scenarios: changes of the impedance due to the variations in the core and shield permeabilities, dependence of the sensor impedance on the plate conductivity of magnetic and nonmagnetic plates and impedance dependence on the sensor lift-off above magnetic and nonmagnetic plates. The numerical implementation of the model is very fast in comparison to the FEM approach (0.7 s vs. 30 min), and this can be improved further if the intermediate results are reused for the next iteration. In comparison with the impedance measurement, the relative error better than 3% in the frequency range from 5 kHz to 100 kHz confirms the applicability of the model for eddy current measurement of material properties and lift-off. The model can be extended easily to include multi-layered planar structures.

## Figures and Tables

**Table 1:** Properties of the sensor used in the numerical studies.

Coil		
Inner radius	$r_{T1}$	2.50 mm
Outer radius	$r_{T2}$	3.15 mm
Bottom position	$z_{T1}$	2.00 mm
Top position	$z_{T2}$	4.00 mm
Lift-off	*h*	0.20 mm
Number of turns	$N_{\mathrm{TX}}$	128
**Core**		
Radius	$r_{C}$	1.75 mm
Height	$L_{C}$	6.00 mm
Rel. permeability	$\mu_{C}$	see the text
**Shield**		
Inner radius	$r_{S1}$	3.65 mm
Outer radius	$r_{S2}$	6.05 mm
Height	$L_{S}$	15.50 mm
Rel. permeability	$\mu_{S}$	see the text

**Table 2:** Properties of the sensor prototype.

Coil		
Inner radius	$r_{T1}$	5.00 mm
Outer radius	$r_{T2}$	5.09 mm
Bottom position	$z_{T1}$	2.45 mm
Top position	$z_{T2}$	17.45 mm
Number of turns	$N_{\mathrm{TX}}$	125
**Core**		
Radius	$r_{C}$	4.98 mm
Height	$L_{C}$	20.03 mm
Material		HF70
Rel. permeability	$\mu_{C}$	1500
**Shield**		
Inner radius	$r_{S1}$	6.45 mm
Outer radius	$r_{S2}$	12.92 mm
Height	$L_{S}$	28.29 mm
Material		4W620
Rel. permeability	$\mu_{S}$	620

**Table 3:** Relative uncertainty of sensor impedance in the case of ±0.01 mm uncertainty in sensor dimensions. The plate has σ = 5 MS/m and $\mu_{r}=50$, lift-off is 0 μm and the frequency is 60 kHz.

Parameter		Value	Uncertainty		Relative Uncertainty of	
				$L_{0}$	ΔR	ΔL
**Coil**						
Inner radius	$r_{T1}$	5.00 mm	±0.01 mm	±2.6 ppm	±2.6 ppm	±2.6 ppm
Outer radius	$r_{T2}$	5.09 mm	±0.01 mm	±2.5 ppm	±2.4 ppm	±2.4 ppm
Bottom position	$z_{T1}$	2.45 mm	±0.01 mm	±542 ppm	∓872 ppm	∓874 ppm
Top position	$z_{T2}$	17.45 mm	±0.01 mm	∓482 ppm	∓856 ppm	∓856 ppm
**Core**						
Radius	$r_{C}$	4.98 mm	±0.01 mm	±6776 ppm	±5632 ppm	±7646 ppm
Height	$L_{C}$	20.03 mm	±0.01 mm	±724 ppm	±879 ppm	±788 ppm
**Shield**						
Inner radius	$r_{S1}$	6.45 mm	±0.01 mm	∓4858 ppm	∓4030 ppm	∓6933 ppm
Outer radius	$r_{S2}$	12.92 mm	±0.01 mm	±8.9 ppm	±30.4 ppm	±65 ppm
Height	$L_{S}$	28.29 mm	±0.01 mm	±0.2 ppm	±0.2 ppm	±0.6 ppm

## Data Availability

The data presented in this study are available on request from the corresponding author.

## Appendix A. Interface Relations for Horizontal Boundaries

The following integrals involving the Bessel functions will be very useful:

(A1) $$\begin{array}{c} \int tX_{\nu }\left(at\right)\Psi_{\nu }\left(bt\right)dt=\pm \frac{t}{a^{2}-b^{2}}\left(aX_{\nu \pm 1}\left(at\right)\Psi_{\nu }\left(bt\right)-bX_{\nu }\left(at\right)\Psi_{\nu \pm 1}\left(bt\right)\right), \end{array}$$

(A2) $$\begin{array}{c} \int tX_{0}\left(at\right)\Psi_{0}\left(at\right)dt=\frac{t^{2}}{2}\left(X_{0}\left(at\right)\Psi_{0}\left(at\right)+X_{1}\left(at\right)\Psi_{1}\left(at\right)\right), \end{array}$$

where *X* and Ψ are any two cylinder functions.

### Appendix A.1. Interface z = L_S_

Because of the piecewise definition of the radial part of the potential, the following equations are obtained from the continuity of $B_{z}$ according to (37):

(A3) $$\begin{array}{c} J_{0}\left(\bar{\alpha }r\right)\exp\left(-\alpha L_{S}\right){\underline{C}}_{1}=\\ \;\;\;\;\;\;=\left\{\begin{array}{c} J_{0}\left(\bar{p}r\right),\;0\leq r\leq r_{S1},\\ R_{0,22}\left(\bar{p}r\right),\;r_{S1}\leq r\leq r_{S2},\\ R_{0,23}\left(\bar{p}r\right),\;r_{S2}\leq r\leq R, \end{array}\right\}\left[\exp\left(-pL_{S}\right){\underline{C}}_{2}+\exp\left(pL_{S}\right){\underline{D}}_{2}\right]. \end{array}$$

If both sides of (A3) are multiplied from the left by the basis function,

(A4) $$X\left(\underline{p}r\right)=\left\{\begin{array}{c} J_{0}\left(\underline{p}r\right),\;0\leq r\leq r_{S1},\\ \frac{1}{\mu_{S}}R_{0,22}\left(\underline{p}r\right),\;r_{S1}\leq r\leq r_{S2},\\ R_{0,23}\left(\underline{p}r\right),\;r_{S2}\leq r\leq R, \end{array}\right.$$

and then integrated from 0 to *R*, we obtain

(A5) $$E\exp\left(-\alpha L_{S}\right){\underline{C}}_{1}=K\exp\left(-pL_{S}\right){\underline{C}}_{2}+K\exp\left(pL_{S}\right){\underline{D}}_{2},$$

where elements of matrices $E=\left(e_{ik}\right)$ and $K=\left(k_{ik}\right)$ are

(A6) $$\begin{array}{c} e_{ik}=\int_{0}^{r_{S1}}rJ_{0}\left(p_{i}r\right)J_{0}\left(\alpha_{k}r\right)dr+\int_{r_{S1}}^{r_{S2}}r\frac{1}{\mu_{S}}R_{0,22}\left(p_{i}r\right)J_{0}\left(\alpha_{k}r\right)dr+\int_{r_{S2}}^{R}rR_{0,23}\left(p_{i}r\right)J_{0}\left(\alpha_{k}r\right)dr=\\ \;\;\;=\frac{1}{p_{i}^{2}-\alpha_{k}^{2}}\left(1-\frac{1}{\mu_{S}}\right)\left[p_{i}r_{S1}J_{1}\left(p_{i}r_{S1}\right)J_{0}\left(\alpha_{k}r_{S1}\right)-p_{i}r_{S2}R_{1,23}\left(p_{i}r_{S2}\right)J_{0}\left(\alpha_{k}r_{S2}\right)\right], \end{array}$$

(A7) $$\begin{array}{c} k_{ik}=\int_{0}^{r_{S1}}rJ_{0}\left(p_{i}r\right)J_{0}\left(p_{k}r\right)dr+\\ +\int_{r_{S1}}^{r_{S2}}r\frac{1}{\mu_{S}}R_{0,22}\left(p_{i}r\right)R_{0,22}\left(p_{k}r\right)dr+\int_{r_{S2}}^{R}rR_{0,23}\left(p_{i}r\right)R_{0,23}\left(p_{k}r\right)dr=\\ =\left\{\begin{array}{c} 0,\;\mathrm{for}\;i\neq \;k\\ \begin{array}{c} \frac{R^{2}}{2}R_{0,23}^{2}\left(p_{i}R\right)+\frac{r_{S2}^{2}}{2}\left(\mu_{S}-1\right)R_{0,23}^{2}\left(p_{i}r_{S2}\right)-\frac{r_{S2}^{2}}{2}\left(1-\frac{1}{\mu_{S}}\right)R_{1,23}^{2}\left(p_{i}r_{S2}\right)-\\ -\frac{r_{S1}^{2}}{2}\left(\mu_{S}-1\right)J_{0}^{2}\left(p_{i}r_{S1}\right)+\frac{r_{S1}^{2}}{2}\left(1-\frac{1}{\mu_{S}}\right)J_{1}^{2}\left(p_{i}r_{S1}\right),\;\mathrm{for}\;i=k, \end{array} \end{array}\right. \end{array}$$

where $p_{i}$ and $\alpha_{k}$ are elements of the vectors $\underline{p}$ and $\bar{\alpha }$, E is full, and K is diagonal.

Continuity of $H_{r}$ according to (38) results in the following:

(A8) $$\begin{array}{c} J_{1}\left(\bar{\alpha }r\right)\exp\left(-\alpha L_{S}\right){\underline{C}}_{1}=\\ =\left\{\begin{array}{c} J_{1}\left(\bar{p}r\right),\;0\leq r\leq r_{S1},\\ \frac{1}{\mu_{S}}R_{1,22}\left(\bar{p}r\right),\;r_{S1}\leq r\leq r_{S2},\\ R_{1,23}\left(\bar{p}r\right),\;r_{S2}\leq r\leq R, \end{array}\right\}\left[\exp\left(-pL_{S}\right){\underline{C}}_{2}-\exp\left(pL_{S}\right){\underline{D}}_{2}\right], \end{array}$$

(A9) $$X\left(\underline{p}r\right)=\left\{\begin{array}{c} J_{1}\left(\underline{p}r\right),\;0\leq r\leq r_{S1},\\ R_{1,22}\left(\underline{p}r\right),\;r_{S1}\leq r\leq r_{S2},\\ R_{1,23}\left(\underline{p}r\right),\;r_{S2}\leq r\leq R, \end{array}\right.$$

(A10) $$F\exp\left(-\alpha L_{S}\right){\underline{C}}_{1}=K\exp\left(-pL_{S}\right){\underline{C}}_{2}-K\exp\left(pL_{S}\right){\underline{D}}_{2},$$

(A11) $$f_{ik}=\frac{1}{p_{i}^{2}-\alpha_{k}^{2}}\left(\mu_{S}-1\right)\left[p_{i}r_{S1}J_{0}\left(p_{i}r_{S1}\right)J_{1}\left(\alpha_{k}r_{S1}\right)-p_{i}r_{S2}R_{0,23}\left(p_{i}r_{S2}\right)J_{1}\left(\alpha_{k}r_{S2}\right)\right].$$

### Appendix A.2. Interface z = L_C_

Continuity of $B_{z}$ according to (37) results in the following:

(A12) $$\begin{array}{c} \left\{\begin{array}{c} J_{0}\left(\bar{p}r\right),\;0\leq r\leq r_{S1},\\ R_{0,22}\left(\bar{p}r\right),\;r_{S1}\leq r\leq r_{S2},\\ R_{0,23}\left(\bar{p}r\right),\;r_{S2}\leq r\leq R, \end{array}\right\}\left[\exp\left(-pL_{C}\right){\underline{C}}_{2}+\exp\left(pL_{C}\right){\underline{D}}_{2}\right]=\\ \;\;\;\;\;=\left\{\begin{array}{c} J_{0}\left(\bar{q}r\right),\;0\leq r\leq r_{C},\\ R_{0,32}\left(\bar{q}r\right),\;r_{C}\leq r\leq r_{S1},\\ R_{0,33}\left(\bar{q}r\right),\;r_{S1}\leq r\leq r_{S2},\\ R_{0,34}\left(\bar{q}r\right),\;r_{S2}\leq r\leq R, \end{array}\right\}\left[\exp\left(-qL_{C}\right){\underline{C}}_{3}+\exp\left(qL_{C}\right){\underline{D}}_{3}\right], \end{array}$$

(A13) $$K\exp\left(-pL_{C}\right){\underline{C}}_{2}+K\exp\left(pL_{C}\right){\underline{D}}_{2}=W\exp\left(-qL_{C}\right){\underline{C}}_{3}+W\exp\left(qL_{C}\right){\underline{D}}_{3},$$

(A14) $$w_{ik}=\frac{1}{p_{i}^{2}-q_{k}^{2}}\left(1-\frac{1}{\mu_{C}}\right)p_{i}r_{C}J_{1}\left(p_{i}r_{C}\right)J_{0}\left(q_{k}r_{C}\right).$$

The basis function $X\left(\underline{p}r\right)$ is given by (A4).

Continuity of $H_{r}$ according to (38) results in the following:

(A15) $$\begin{array}{c} \left\{\begin{array}{c} J_{1}\left(\bar{p}r\right),\;0\leq r\leq r_{S1},\\ \frac{1}{\mu_{S}}R_{1,22}\left(\bar{p}r\right),\;r_{S1}\leq r\leq r_{S2},\\ R_{1,23}\left(\bar{p}r\right),\;r_{S2}\leq r\leq R, \end{array}\right\}\left[\exp\left(-pL_{C}\right){\underline{C}}_{2}-\exp\left(pL_{C}\right){\underline{D}}_{2}\right]=\\ \;\;\;\;=\left\{\begin{array}{c} \frac{1}{\mu_{C}}J_{1}\left(\bar{q}r\right),\;0\leq r\leq r_{C},\\ R_{1,32}\left(\bar{q}r\right),\;r_{C}\leq r\leq r_{S1},\\ \frac{1}{\mu_{S}}R_{1,33}\left(\bar{q}r\right),\;r_{S1}\leq r\leq r_{S2},\\ R_{1,34}\left(\bar{q}r\right),\;r_{S2}\leq r\leq R, \end{array}\right\}\left[\exp\left(-qL_{C}\right){\underline{C}}_{3}-\exp\left(qL_{C}\right){\underline{D}}_{3}\right], \end{array}$$

(A16) $$K\exp\left(-pL_{C}\right){\underline{C}}_{2}-K\exp\left(pL_{C}\right){\underline{D}}_{2}=L\exp\left(-qL_{C}\right){\underline{C}}_{3}-L\exp\left(qL_{C}\right){\underline{D}}_{3},$$

(A17) $$l_{ik}=\frac{1}{p_{i}^{2}-q_{k}^{2}}\left(1-\frac{1}{\mu_{C}}\right)p_{i}r_{C}J_{0}\left(p_{i}r_{C}\right)J_{1}\left(q_{k}r_{C}\right).$$

In this case, the basis function $X\left(\underline{p}r\right)$ is given by (A9).

### Appendix A.3. Interface z = z_0_

Continuity of $B_{z}$ according to (37) results in the following:

(A18) $$\exp\left(-qz_{0}\right){\underline{C}}_{3}+\exp\left(qz_{0}\right){\underline{D}}_{3}=\exp\left(-qz_{0}\right){\underline{C}}_{4}+\exp\left(qz_{0}\right){\underline{D}}_{4}.$$

Continuity of $H_{r}$ according to (38) results in the following:

(A19) $$\begin{array}{c} \left\{\begin{array}{c} \frac{1}{\mu_{C}}J_{1}\left(\bar{q}r\right),\;0\leq r\leq r_{C},\\ R_{1,32}\left(\bar{q}r\right),\;r_{C}\leq r\leq r_{S1},\\ \frac{1}{\mu_{S}}R_{1,33}\left(\bar{q}r\right),\;r_{S1}\leq r\leq r_{S2},\\ R_{1,34}\left(\bar{q}r\right),\;r_{S2}\leq r\leq R, \end{array}\right\}\left[\exp\left(-qz_{0}\right){\underline{C}}_{3}-\exp\left(qz_{0}\right){\underline{D}}_{3}\right]=\\ \left\{\begin{array}{c} \frac{1}{\mu_{C}}J_{1}\left(\bar{q}r\right),\;0\leq r\leq r_{C},\\ R_{1,42}\left(\bar{q}r\right),\;r_{C}\leq r\leq r_{S1},\\ \frac{1}{\mu_{S}}R_{1,43}\left(\bar{q}r\right),\;r_{S1}\leq r\leq r_{S2},\\ R_{1,44}\left(\bar{q}r\right),\;r_{S2}\leq r\leq R, \end{array}\right\}\left[\exp\left(-qz_{0}\right){\underline{C}}_{4}-\exp\left(qz_{0}\right){\underline{D}}_{4}\right]+\mu_{0}I\delta \left(r-r_{0}\right), \end{array}$$

(A20) $$X\left(\underline{q}r\right)=\left\{\begin{array}{c} J_{1}\left(\underline{q}r\right),\;0\leq r\leq r_{C},\\ R_{1,32}\left(\underline{q}r\right),\;r_{C}\leq r\leq r_{S1},\\ R_{1,33}\left(\underline{q}r\right),\;r_{S1}\leq r\leq r_{S2},\\ R_{1,34}\left(\underline{q}r\right),\;r_{S2}\leq r\leq R, \end{array}\right.$$

(A21) $$\begin{array}{c} \exp\left(-qz_{0}\right){\underline{C}}_{3}-\exp\left(qz_{0}\right){\underline{D}}_{3}=\\ \;\;\;\;\;\;\;\;\;\;=\exp\left(-qz_{0}\right){\underline{C}}_{4}-\exp\left(qz_{0}\right){\underline{D}}_{4}+\mu_{0}IS^{-1}r_{0}R_{1,32}\left(\underline{q}r_{0}\right), \end{array}$$

(A22) $$s_{ik}=\left\{\begin{array}{c} 0,\;\mathrm{for}\;i\neq k\\ \begin{array}{c} \frac{R^{2}}{2}R_{0,34}^{2}\left(q_{i}R\right)+\frac{r_{C}^{2}}{2}\frac{1}{\mu_{C}}\left(1-\frac{1}{\mu_{C}}\right)J_{0}^{2}\left(q_{i}r_{C}\right)-\frac{r_{C}^{2}}{2}\left(1-\frac{1}{\mu_{C}}\right)J_{1}^{2}\left(q_{i}r_{C}\right)-\\ -\frac{r_{S1}^{2}}{2}\frac{1}{\mu_{S}}\left(1-\frac{1}{\mu_{S}}\right)R_{0,33}^{2}\left(q_{i}r_{S1}\right)+\frac{r_{S1}^{2}}{2}\left(1-\frac{1}{\mu_{S}}\right)R_{1,33}^{2}\left(q_{i}r_{S1}\right)+\\ +\frac{r_{S2}^{2}}{2}\left(\mu_{S}-1\right)R_{0,34}^{2}\left(q_{i}r_{S2}\right)-\frac{r_{S2}^{2}}{2}\left(1-\frac{1}{\mu_{S}}\right)R_{1,34}^{2}\left(q_{i}r_{S2}\right),\;\mathrm{for}\;i=k. \end{array} \end{array}\right.$$

### Appendix A.4. Interface z = 0

Continuity of $B_{z}$ according to (37) results in the following:

(A23) $$\left\{\begin{array}{c} J_{0}\left(\bar{q}r\right),\;0\leq r\leq r_{C},\\ R_{0,42}\left(\bar{q}r\right),\;r_{C}\leq r\leq r_{S1},\\ R_{0,43}\left(\bar{q}r\right),\;r_{S1}\leq r\leq r_{S2},\\ R_{0,44}\left(\bar{q}r\right),\;r_{S2}\leq r\leq R, \end{array}\right\}\left({\underline{C}}_{4}+{\underline{D}}_{4}\right)=J_{0}\left(\bar{\alpha }r\right)\left({\underline{C}}_{5}+{\underline{D}}_{5}\right),$$

(A24) $$X\left(\underline{q}r\right)=\left\{\begin{array}{c} \frac{1}{\mu_{C}}J_{0}\left(\underline{q}r\right),\;0\leq r\leq r_{C},\\ R_{0,32}\left(\underline{q}r\right),\;r_{C}\leq r\leq r_{S1},\\ \frac{1}{\mu_{S}}R_{0,33}\left(\underline{q}r\right),\;r_{S1}\leq r\leq r_{S2},\\ R_{0,34}\left(\underline{q}r\right),\;r_{S2}\leq r\leq R, \end{array}\right.$$

(A25) $$S{\underline{C}}_{4}+S{\underline{D}}_{4}=V{\underline{C}}_{5}+V{\underline{D}}_{5},$$

(A26) $$\begin{array}{c} v_{ik}=\frac{1}{q_{i}^{2}-\alpha_{k}^{2}}\left[-\left(1-\frac{1}{\mu_{C}}\right)q_{i}r_{C}J_{1}\left(q_{i}r_{C}\right)J_{0}\left(\alpha_{k}r_{C}\right)+\right.\\ \left.+\left(1-\frac{1}{\mu_{S}}\right)q_{i}r_{S1}R_{1,43}\left(q_{i}r_{S1}\right)J_{0}\left(\alpha_{k}r_{S1}\right)-\right.\\ \left.-\left(1-\frac{1}{\mu_{S}}\right)q_{i}r_{S2}R_{1,44}\left(q_{i}r_{S2}\right)J_{0}\left(\alpha_{k}r_{S2}\right)\right]. \end{array}$$

Continuity of $H_{r}$ according to (38) results in the following:

(A27) $$\left\{\begin{array}{c} \frac{1}{\mu_{C}}J_{1}\left(\bar{q}r\right),\;0\leq r\leq r_{C},\\ R_{1,42}\left(\bar{q}r\right),\;r_{C}\leq r\leq r_{S1},\\ \frac{1}{\mu_{S}}R_{1,43}\left(\bar{q}r\right),\;r_{S1}\leq r\leq r_{S2},\\ R_{1,44}\left(\bar{q}r\right),\;r_{S2}\leq r\leq R, \end{array}\right\}\left({\underline{C}}_{4}-{\underline{D}}_{4}\right)=J_{1}\left(\bar{\alpha }r\right)\left({\underline{C}}_{5}-{\underline{D}}_{5}\right),$$

(A28) $$S{\underline{C}}_{4}-S{\underline{D}}_{4}=U{\underline{C}}_{5}-U{\underline{D}}_{5},$$

(A29) $$\begin{array}{c} u_{ik}=\frac{1}{q_{i}^{2}-\alpha_{k}^{2}}\left[-\left(1-\frac{1}{\mu_{C}}\right)q_{i}r_{C}J_{0}\left(q_{i}r_{C}\right)J_{1}\left(\alpha_{k}r_{C}\right)+\right.\\ \;\;\;\;\;\;\;\;\;\left.+\left(1-\frac{1}{\mu_{S}}\right)q_{i}r_{S1}R_{0,43}\left(q_{i}r_{S1}\right)J_{1}\left(\alpha_{k}r_{S1}\right)-\right.\\ \;\;\;\;\;\;\;\;\;\;\;\;\;\;\left.-\left(\mu_{S}-1\right)q_{i}r_{S2}R_{0,44}\left(q_{i}r_{S2}\right)J_{1}\left(\alpha_{k}r_{S2}\right)\right]. \end{array}$$

The basis function $X\left(\underline{q}r\right)$ is given by (A20).

### Appendix A.5. Interface z = −h

Continuity of $B_{z}$ and $H_{r}$ according to (37) and (38) result in the following:

(A30) $$\exp\left(\alpha h\right){\underline{C}}_{5}+\exp\left(-\alpha h\right){\underline{D}}_{5}=\alpha \beta^{-1}\exp\left(-\beta h\right){\underline{D}}_{6},$$

(A31) $$\exp\left(\alpha h\right){\underline{C}}_{5}-\exp\left(-\alpha h\right){\underline{D}}_{5}=-\frac{1}{\mu_{r}}\exp\left(-\beta h\right){\underline{D}}_{6}.$$
